# Machine learning demonstrates clinical utility in distinguishing retinoblastoma from pseudo retinoblastoma with RetCam images

**DOI:** 10.1080/13816810.2025.2455576

**Published:** 2025-01-20

**Authors:** Owen Cruz-Abrams, Ricardo Dodds Rojas, David H. Abramson

**Affiliations:** a Department of Surgery, Memorial Sloan Kettering Cancer Center, New York, N.Y, US; b DigITs, Memorial Sloan Kettering Cancer Center, New York, New York, USA

**Keywords:** Retinoblastoma, coats disease, AI artificial learning, ML machine learning, RetCam, ResNet, enucleation

## Abstract

**Background:**

Retinoblastoma is diagnosed and treated without biopsy based solely on appearance (with the indirect ophthalmoscope and imaging). More than 20 benign ophthalmic disorders resemble retinoblastoma and errors in diagnosis continue to be made worldwide. A better noninvasive method for distinguishing retinoblastoma from pseudo retinoblastoma is needed.

**Methods:**

RetCam imaging of retinoblastoma and pseudo retinoblastoma from the largest retinoblastoma center in the U.S. (Memorial Sloan Kettering Cancer Center, New York, NY) were used for this study. We used several neural networks (ResNet-18, ResNet-34, ResNet-50, ResNet-101, ResNet-152, and a Vision Image Transformer, or VIT), using 80% of images for training, 10% for validation, and 10% for testing.

**Results:**

Two thousand eight hundred eighty-two RetCam images from patients with retinoblastoma at diagnosis, 1,970 images from pseudo retinoblastoma at diagnosis, and 804 normal pediatric fundus images were included. The highest sensitivity (98.6%) was obtained with a ResNet-101 model, as were the highest accuracy and F1 scores of 97.3% and 97.7%. The highest specificity (97.0%) and precision (97.0%) was attained with a ResNet-152 model.

**Conclusion:**

Our machine learning algorithm successfully distinguished retinoblastoma from retinoblastoma with high specificity and sensitivity and if implemented worldwide will prevent hundreds of eyes from incorrectly being surgically removed yearly.

## 1. Introduction

Intraocular tumors and retinoblastoma in particular are routinely diagnosed and treated without biopsy (1) because biopsy and intraocular surgery on eyes with retinoblastoma (unsuspected) can result in extraocular extension and death (2). Because of that, retinoblastoma is routinely diagnosed with the indirect ophthalmoscope and confirmed with retinal imaging (usually the RetCam system) (3). Ancillary imaging is often used but notoriously nonspecific. CT scans are no longer used (because of the ionizing radiation) and while ultrasound and MRI are sometimes helpful, they are fraught with false positives and negatives (1–5).

Many intraocular lesions can look like retinoblastoma, and the differential diagnosis includes more than 20 separate conditions (4). In the 20th century errors were common (6, 7). Despite improvement in ultrasound, MR and retinal imaging, errors continue to occur. The incidence of errors worldwide is not known, but recent series suggest an error rate of 10% in some countries (8–16).

The use of machine learning in medicine has exploded in the past 10 years. In ophthalmology, it has been investigated extensively for retinal diseases, including diabetes (17), retinal detachment (18), macular degeneration (19), macula holes (20), central retinal vein occlusion (21), retinitis pigmentosa (22), retinopathy of prematurity (23) reticular pseudodrusen (24) utilizing fundus photography, and OCT and fluorescein angiography (25). It has also been used for glaucoma (26), cataract (27), oculoplastics (28), keratoconus (29), refractive surgery (30), and strabismus surgery (31).

Machine learning has also been used in retinoblastoma. It has been used with external photographs to detect leukocoria (32) in addition to differentiating a retinoblastoma fundus from a normal fundus (33) and also used as a model for the economic implications of ML (34) but has not been used not to distinguish retinoblastoma from pseudo retinoblastoma. For this study, we developed a machine learning (ML) algorithm to differentiate retinoblastoma cases from pseudo retinoblastoma cases based on a single RetCam image.

## 2. Materials and methods

This is a single institution MSKCC IRB approved study based on clinical imaging from the Ophthalmic Oncology Service that was done with the RetCam digital imaging system. We selected de-identified images from the service’s images, specifically those of retinoblastoma and pseudo retinoblastoma and normal pediatric eyes, excluding any patient that had received any form of ocular treatment including radiation, chemotherapy, laser, cryotherapy, or surgery (because treated tumors have a different appearance from naïve, untreated tumors). We excluded external photographs but did include close-up RetCam photographs including iris images if there was useful fundus imaging. Photographs that were not of the eye (such as photographs of equipment) were excluded. All images had a 4:3 aspect ratio and were of size 1600 × 1200px or 640 × 480px. Except for these exclusions no case of pseudoretinoblastoma was excluded.

To create a dataset suitable for model training and inference, we augmented the images with a random horizontal flip. We further applied black-pixel padding to all of the images to make them square, and resized them to 224 × 224px (standard ResNet input size) (35).

We fine-tuned several neural networks (ResNet-18, ResNet-34, ResNet-50, ResNet-101, ResNet-152 (36), and a Vision Image Transformer, or VIT (37), using 80% of images for training, 10% for validation, and 10% for testing (splitting into folders randomly). We created two separate datasets for fine-tuning, one with only retinoblastoma and pseudo retinoblastoma eyes, and one with those and normal pediatric eyes. We included the normal eyes under “not retinoblastoma” but used the same test dataset (the one without normal images) for both sets of models to determine if the inclusion of normal images in only the training and validation sets affected model performance on abnormal eyes.

## 3. Results

We selected 5,566 RetCam fundus images from the Ophthalmic Oncology Service at MSKCC. Of these, 2,882 were of retinoblastomas, while 1,970 were of pseudo retinoblastomas and 804 normals. These pseudo retinoblastoma cases include patients with Coats Disease, Persistent Hyperplastic Primary Vitreous (PHPV)/Persistent Fetal Vasculature Syndrome (PFV), Cataract, Toxoplasmosis, Nevus of Ota, Tuberous sclerosis, Morning Glory syndrome, Microphthalmos, Pseudoglioma, Myelinated nerve fibers, Persistent Burgmeisters Papilla, Falciform fold, Epithelial cyst, Anterior segment dysgenesis, optic atrophy, Incontinentia pigmenti, chorioretinal scar, CMV retinitis, optic chiasmatic tumor, retinal hemorrhage, vitreous hemorrhage, papilledema, and dysplastic retina. Using an 80-10-10 split, we created a dataset containing: 2,223 retinoblastoma images and 1,576 pseudo retinoblastoma images (plus 541 normal images) for training, 280 retinoblastoma images and 197 pseudo retinoblastoma images (plus 197 normal images) for validation, and 276 retinoblastoma images and 198 pseudo retinoblastoma images for testing. We trained ResNets in parallel on these data using the hyperparameters listed below.

Hyperparameter values:

- 25 Epochs
- Learning Rate Decay—step of 0.1 every 7 epochs
- Optimizer: SGD (Stochastic Gradient Descent) with a momentum of 0.9
- Pre-trained weights: ImageNet-1K
- Batch Size: 32 images/batch
- Learning Rate: 1 × 10^−3^
- Loss function: CrossEntropyLoss (Crossentropy)
- VIT Base Model: vit_base_patch16_224

**Figure 1.**
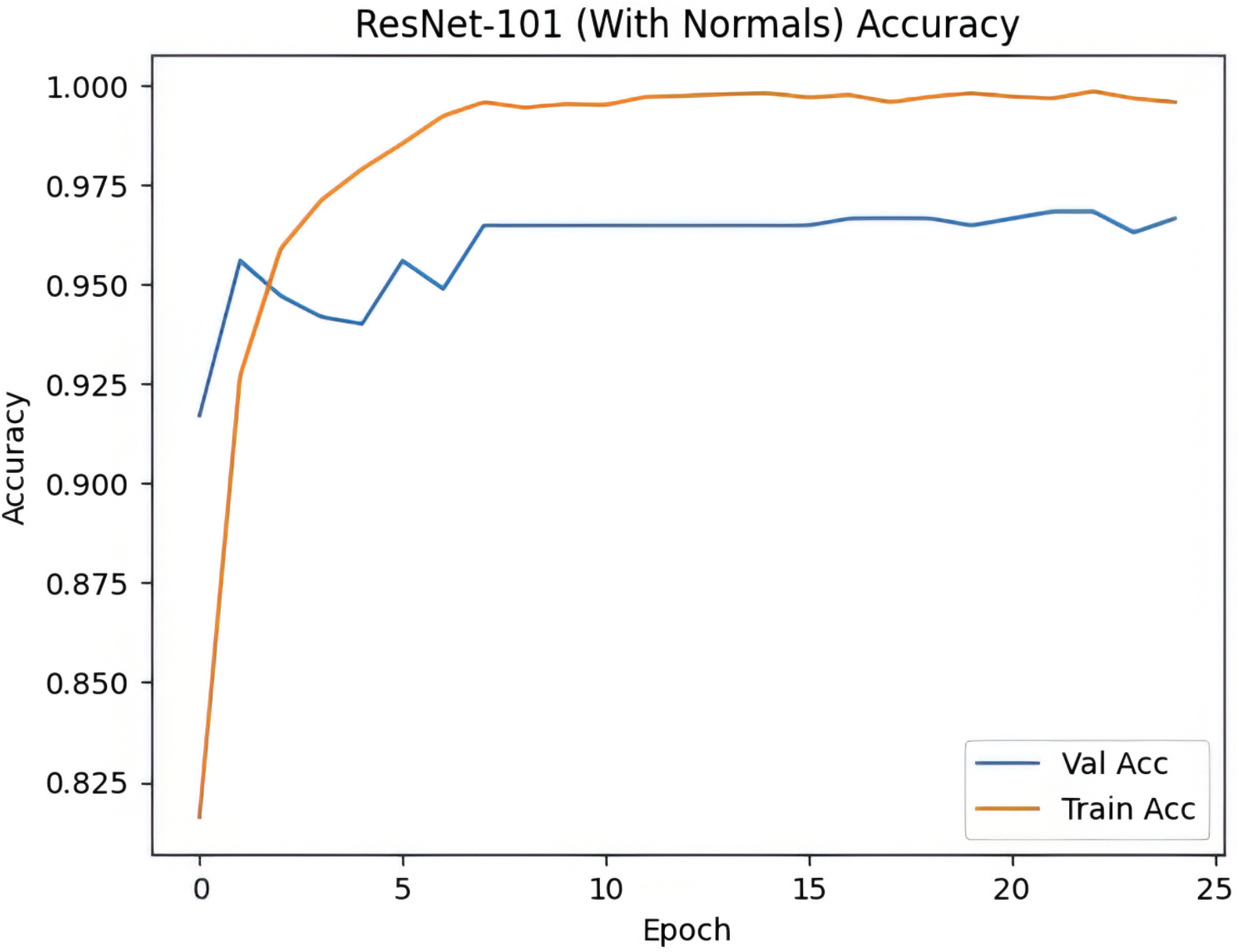
Model training history.

**Figure 2.**
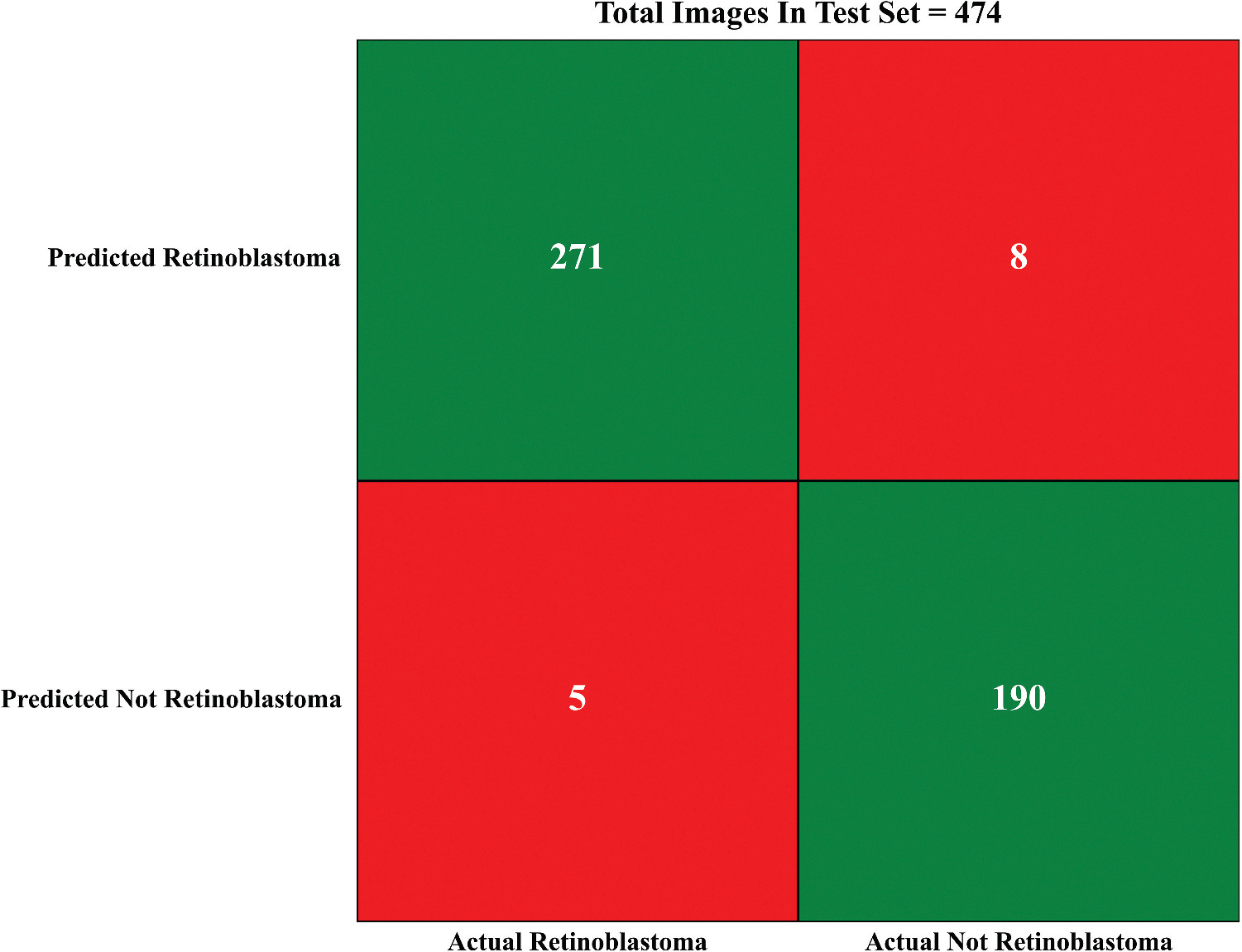
Confusion matrix ResNet-101 with normals.

The summary of our model’s performance on the test set is listed in Table 1; the summary of the most accurate model’s is presented in Figure 1. The confusion matrix of our best model is shown in Figure 2.

## 4. Discussion

We have developed an algorithm that utilizes RetCam imaging and enables clinicians to distinguish retinoblastoma from lesions that simulate retinoblastoma. While the diagnosis of retinoblastoma is often straightforward in experienced centers, errors continue to be made by clinicians worldwide. For example, here is a (partial) list of published incorrect diagnoses made in the past 11 years: granulomatous endophthalmitis, retinal astrocytoma, Coats disease, PHPV, retinal dysplasia, colobomas, retinal detachment, optic disc hypoplasia, optic nerve drusen, combined hamartoma, retinopathy of prematurity, FEVR, vitreous hemorrhage, and congenital glaucoma (8–16). Despite the use of ultrasound, CT and MR imaging these errors still occur (and this knowledge is based on published series-it is suspected that many more errors are happening but for obvious reasons these centers do not publish their errors.

Machine learning and AI have been used before in retinoblastoma. For example, Aldughayfiq and colleagues developed a deep learning model using an established dataset of 400 retinoblastoma images and 400 normal fundus images (38). They demonstrated an accuracy of 97% on the test set. Using LIME (local interpretable model-agnostic explanations) and SHAP (SHapley Additive exPlanations) they demonstrated that the model could reliably differentiate a fundus image of retinoblastoma from a normal fundus image. Their “normal fundus images” were taken from an established library of normal *adult* eyes. Retinoblastoma only develops in children’s eyes (with very rare exceptions) so their control normal eyes are of interest in developing a model but not useful for clinicians. In clinical practice, differentiating a normal eye from an eye with retinoblastoma is easy and not the clinical challenge. More than 20 different conditions can simulate the fundus appearance of retinoblastoma (Coats disease being the most common) and the clinical challenge and need for improvement are differentiating retinoblastoma from these diverse nonmalignant conditions.

Kaliki et al. did something similar (33). They created an AI model based on 771 fundus images of 109 eyes. Five hundred ninety demonstrated retinoblastoma and 181 had no tumor. The sensitivity was 85%, the specificity 99%. Pseudo retinoblastomas were not included. Their “normal” were the fellow eye of unilateral retinoblastoma patients (children). Their dataset was mostly from heavily pigmented eyes, so its veracity in lightly pigmented eyes is unknown.

A number of teams have developed useful algorithms to detect leukocoria based on external photographs. For example, Munson and colleagues developed a mobile application based on 52,982 external photographs of 40 children (8 with retinoblastoma) with a reported sensitivity of 90% (32). RetCam images were not used. This tool was designed to detect leukocoria (including retinoblastoma) but not differentiate retinoblastoma from pseudo retinoblastoma. A recent paper on 210 eyes emphasized that “AI models built on a single race do not work well for other races” (39). This is an important feature of AI algorithms and for a scheme to work worldwide a challenge that must be addressed.

In a recent meta-analysis of the literature on ASI/ML in retinoblastoma (40) it was concluded that only 6 studies met criteria and that … ”Given the notorious reduction in the number of pediatric ophthalmologists … and the overall difficulty in accessing ocular oncologists …, AI emerges as a compelling and sensitive screening strategy that could serve as a bridge to specialists in places with scarcity of them”.

Of the 12 models included in our study, the highest accuracy was 97.3%, Sensitivity of 98.6%, Specificity of 97%, F1 score of 97.7% and Precision of 97% with ResNet outperforming the Vit in all categories.

Our study differs as we compared fundus images of retinoblastoma patients who had not received any treatment to fundus images of pseudo retinoblastoma that had also not had prior treatment. Our goal was to create a useful, simple to use, reliable algorithm that would aid clinicians in the differential diagnosis of retinoblastoma. Errors in diagnosis can be fatal. If a child with retinoblastoma is thought to have a benign lesion (and therefore not treated) death can ensue.

**Figure 3.**
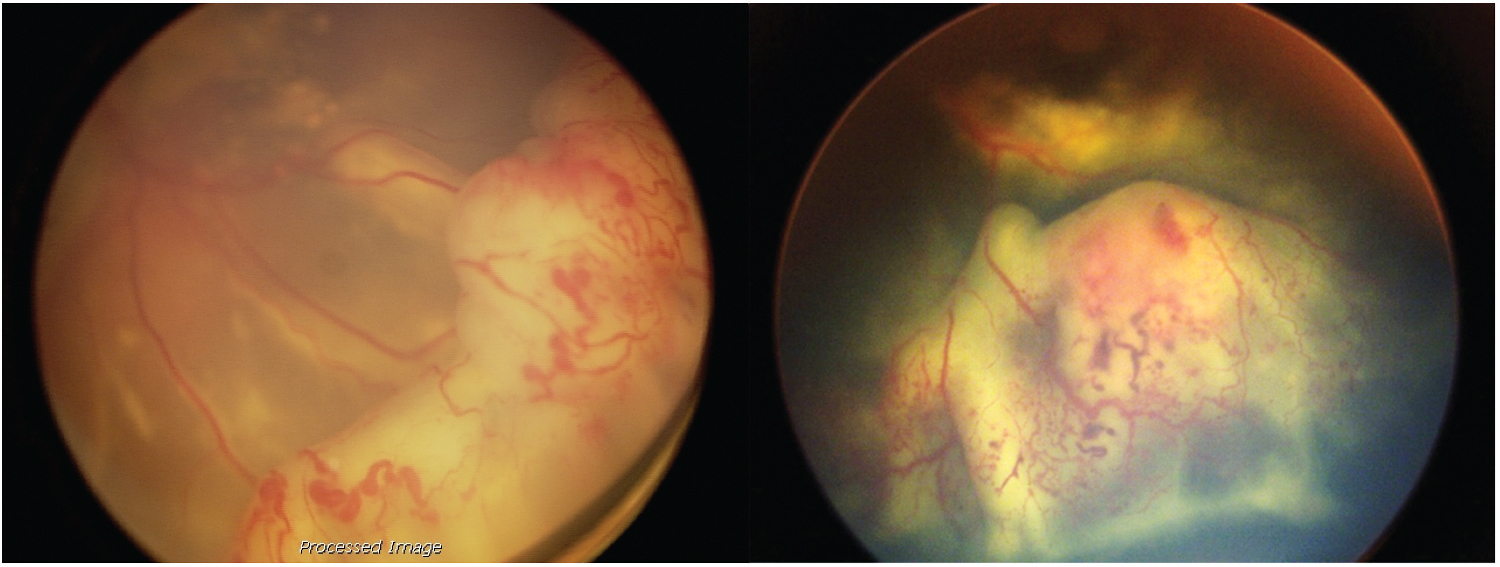
Benign lesions simulating retinoblastoma (Coats disease).

If a child with a benign lesion (possibly treatable) is thought to have retinoblastoma and receives radiation, chemotherapy or enucleation loss of an eye or vision-to say nothing about treatment complications which are acceptable if the eye had contained cancer (Figure 3).

The true incidence of errors in retinoblastoma diagnosis in 2024 is not known, but some recent series suggest an error rate of 10% in some countries (8–10). Using our algorithm may help minimize incorrect diagnoses and treatments. Most models improve with larger numbers and our database of over 30,000 images and those of other groups will improve on our model.

**Figure 4.**
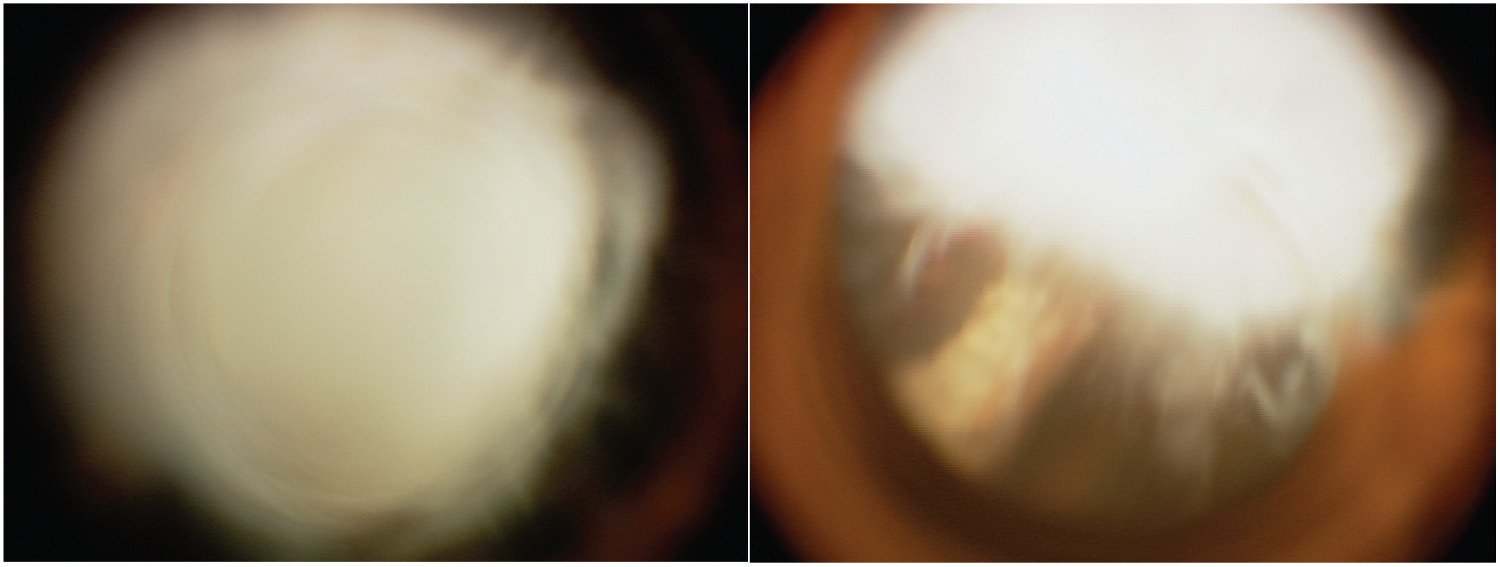
Pseudo retinoblastoma incorrectly identified as retinoblastoma by our algorithm (left) but correctly identified as benign retrolenticular lesion by the model on additional imaging (right).

We also learned about some of the limitations of this approach. Retinoblastomas in the eye are three-dimensional structures, and RetCam images are two-dimensional entities. An example of an error our algorithm made is presented in Figure 4. In Figure 4 what appears to be an elevated white/creamy mass in the retina was actually a benign fibrovascular lesion just behind the lens. The model misinterpreted the first image but correctly identified the second (as well as all other images from that eye, which were in the training set) as pseudo retinoblastoma. This diagnosis was easily appreciated by the clinician using the indirect ophthalmoscope (which allows the viewer a 3-D view), thus we expect clinicians would use our algorithm in conjunction with indirect ophthalmoscopy.

We also recognized that the “field of view” seen in a RetCam image influenced results. In some cases, a RetCam image from an eye will not show the tumor as it may be outside the field captured by the photograph. Our algorithm included all images taken from eyes with retinoblastoma, but we did not filter out images where there was no visible tumor. Presumably, when our algorithm is used by clinicians, they will only submit images that demonstrate pathology, eliminating this source of error.

## Figures and Tables

**Table 1.** Lists model performance on the test set for each of the models we trained.

Model	Accuracy	Sensitivity/Recall	Specificity	F1 Score	Precision

ResNet-18	94.7%	97.1%	91.4%	95.5%	91.9%
ResNet-18 with normals	96.4%	97.1%	95.5%	97.0%	95.5%
ResNet-34	96.2%	96.70%	95.50%	96.70%	95.5%
ResNet-34 with normals	96.6%	97.1%	96.0%	97.1%	96.0%
ResNet-50	96.4%	97.1%	95.5%	96.9%	95.5%
ResNet-50 with normals	95.6%	94.9%	96.5%	96.1%	96.4%
ResNet-101	95.8%	***98.6%***	91.9%	96.5%	92.4%
ResNet-101 with normals	***97.3%***	98.2%	96.0%	***97.7%***	96.0%
ResNet-152	96.6%	96.4%	***97.0%***	97.1%	***97.0%***
ResNet-152 with normals	96.6%	96.7%	96.5%	97.1%	96.5%
VIT	78.1%	84.4%	69.2%	81.8%	79.3%
VIT with normals	88.4%	88.8%	87.9%	90.0%	91.1%

The number that was the highest number in each category is listed in ***bold/italics***.
